# Fatigue and performance in interpreting breast screening mammograms

**DOI:** 10.1186/bcr3279

**Published:** 2012-11-09

**Authors:** S Taylor-Phillips, A Clarke, M Wheaton, O Kearins, M Wallis

**Affiliations:** 1 The University of Warwick, Coventry, UK; 2 University Hospital Coventry and Warwickshire, Coventry, UK; 3 West Midlands Quality Assurance Reference Centre, Birmingham, UK; 4 Addenbrookes Hospital, Cambridge, UK

## Introduction

Interpreting mammograms is a repetitive visual task, which may cause fatigue. Current practice in England for digital mammography is that both readers examine batches of mammograms in the same order as one another. This research examines whether there is a potential benefit in the two readers examining the cases in a different order to one another, to ameliorate any effects of fatigue at the system level.

## Methods

NBSS records at one screening centre for 4 years were examined (2007 to 2010, seven readers, >170,000 cases). The time and date that each case was reported was extracted from NBSS. A period of over 1 hour without reporting any cases was defined as a break. Recall rate was compared for the first 10 cases since a break and the 10 cases after that using a within-subjects *t *test.

## Results

Each reader examined between 20,080 and 74,028 cases over 4 years, and recall rates ranged from 3.6% to 5.9%. Recall rate was 2.3% higher for the first 10 cases after the break than for the subsequent 10 cases (*P *= 0.004). The sample was too small to examine effects on cancer detection rate.

## Conclusion

There may be patterns in performance with time since a break. Further research is needed to ascertain whether these patterns remain present in a larger more controlled sample, and whether changing case order could improve overall performance. The Changing case Order to Optimise patterns of Performance in Screening (CO-OPS) randomised controlled trial will be begin recruitment in England soon to address these questions.

